# Biographical Memoir of Mr. Wm. Tebb

**Published:** 1899-07

**Authors:** M. R. Leverson

**Affiliations:** Fort Hamilton, L. I., N. Y.


### BIOGRAPHICAL MEMOIR OF MR. WM. TEBB.

M. R. Leverson, M. D., Fort Hamilton, L. I., N. Y.

(Continued from June No., p. 287.)

The election of Abraham Lincoln as President of the United States was justly regarded as a triumph for the Anti-Slavery party and created intense excitement throughout the country. A few days after the election Mr. Tebb was on his way to St. Louis, a city where violence had been repeatedly shown to persons suspected of being inimical to the slave power. After remaining in the city a few days he took the train to Kansas, where Free State colonies with much difficulty had been established and defended by riflemen with arms supplied by the Free State party in the New England States. When in the cars he happened to sit next to a young man who was traveling in the same direction, and conversation turned on the political situation. The conversation was overheard; a meeting of Missourians was rapidly convened, and the resolution was arrived at of ejecting them from the train and throwing them into the Missouri river which ran close by. This was in the days of what was known as "border ruffianism," when raids and outrages upon Free State settlers were of every-day occurrences. Looking at these miscreants, Mr. Tebb felt as though he was in a cage of wild beasts, and would undoubtedly have been sacrificed to their fury had it not been that in the midst of the excitement and contemplated outrage, the train pulled up at the vine growing settlement of Hermann occupied by a German community. By means of the friendly aid of the train conductor, and amidst the confusion of passengers getting in and out of the train he succeeded in escaping and took refuge in a wine shop until the train had passed on. After walking through the village he took the first train back to St. Louis, and the steamer down the Mississippi to Cairo, Illinois.

In 1862, his health having again become undermined by excessive labors and by the malarial fevers common to so many places in the United States, to which the lowering of his vitality by venesection rendered him particularly susceptible, Mr. Tebb returned to England. There his business capacity soon found employment. He became interested in and a director of a company established for the manufacture of chlorine and of a bleaching powder by new processes which were destined to revolutionize their manufacture and to cheapen every sheet of paper throughout the world. The patents have, however, long since expired.

In 1870 Mr. Tebb's attention was drawn to the subject of vaccination in the manner so graphically described in his testimony before the Royal (British) Commission, on the 16th of May, 1890. (See Q. 9,457.) As an abstract thereof is given in The Homœopathic Physician, September, 1898, page 384, it will not be repeated here.

The repeated prosecutions to which Mr. Tebb was subjected for refusing to have his children vaccinated, opened his eyes to the cruel iniquity of the law, and had the effect of leading him to make a thorough investigation of the question of vaccination from every point of view, and has been of infinite value to the public health and to human rights, violated in their most fundamental principles by law-enforced vaccination over the greater part of the civilized world. But for the repeated persecution to which Mr. Tebb was subjected, it may well be doubted whether his attention would have been called to the monstrous character of those laws, and the cause might have lacked the support of his indomitable energy and admirable skill in conducting the struggle, as also the substantial support furnished by himself, the Countess de Noailles, the late Mr. P. A. Taylor, and other friends of personal freedom. That without this financial backing the cause would most likely have languished on for many more years, is rendered probable by its condition in the United States at the present time, where just such support is lacking.

Mr. Tebb has traveled into every portion of the globe in prosecution of his researches into the effects of vaccination. Among the countries visited were every part of his own country from Land's End to the Shetland Islands, almost every State in Europe from the Mediterranean to the North Cape, countries intervening between the Tagus in the west and the Volga, Danube, and Bosphorus in the east; Morocco, Algeria, Upper and Lower Egypt, Asia Minor, Upper and Lower Canada, Nova Scotia, and most of the States and Territories of the United States; India, China, Japan, Venezuela and British Guiana, the Virgin, Windward, and Leeward Islands, the French and Danish West Indies, the Archipelagoes of Greece and Hawaii, the Island of Ceylon, Tasmania, New Zealand, and the colonies of Australia.

In an interview which Mr. Tebb recently gave to a reporter from the Boston *Evening Transcript*, he said:

"One of my principal efforts in visiting some of these countries has been devoted to investigating the alleged increase of leprosy in various parts of the world, a subject which has peculiar significance to the United States now that it has extended its boundaries so as to include countries that are afflicted with this disease. I have recently published a volume of over four hundred pages entitled **The Recrudescence of*

---

*A copy of this work presented by the author to his wife bears the following inscription:

"To my dear wife, who first called my attention to the evils of vaccination; who stood by my side during thirteen harassing police court vaccination prosecutions; who for more than twenty years has been the devoted companion of my labors in the arduous struggle for the emancipation of my countrymen from an odious and indefensible tyranny, and who has

*Leprosy and its Causation*, giving the detailed results of my investigation, including the testimony of upwards of over forty physicians and medical superintendents of leper hospitals, showing that leprosy is an inoculable disease and is communicable by means of a cut, bruise, abraded surface sore, and principally by vaccine virus taken from a leprous subject. The population of the Sandwich Islands, which, when discovered by Captain Cook, was estimated at half a million, now numbers only about twenty-five thousand natives, and the diminution continues, partly by reason of the increased death rate due to leprosy caused by vaccination. The result of my inquiries may be briefly summarized as follows:

"That evidence from all authorities shows that leprosy is seriously increasing throughout the West Indies, that the theory of contagion put forward to account for this increase is doubtful, and is denied by the highest medical authorities, that the only method of inoculation extensively practiced is by means of arm-to-arm vaccination, and that leprosy has been distinctly traced to this source by medical practitioners in the West Indies and in Norway, including several medical superintendents of leper asylums, and by distinguished authorities such as Dr. Tilbury Fox, Sir Erasmus Wilson, Dr. Gavin Milroy, Prof. W. T. Gardiner of Glasgow, Dr. G. A. Hansen, superintendent of the leper asylum at Bergen, Dr. A. M. Brown, Dr. Hall Bakewell, Dr. Bechtinger, and others. A well known medical practitioner of Honolulu unhesitatingly expressed the opinion that the dissemination of leprosy in Hawaii was largely due to inoculation by the lancet of the public vaccinator, a most serious matter not only for Hawaii, but for all other countries where the repulsive and destructive disease is epidemic.

---

accompanied and encouraged me in the researches in various parts of the globe detailed herein, I present this first bound copy of a work to which her inspiration has so largely contributed.

"BARSTOW, SURREY, Jan. 23d, 1898."

"When in India I made extensive inquiries regarding the increase of leprosy, which I attribute to the same cause—vaccination. Mr. H. Brown of Simla, who has devoted much attention to the subject, wrote in 1889:

" 'Leprosy is increasing in India, almost by leaps and bounds, and the Government, disinclined as it has hitherto been to give the subject attention, is now obliged to move in the matter. The present number of lepers in India has been calculated at three hundred thousand, but are probably twice as many. A new home for lepers has just been opened at Matoonga, near Bombay, with accommodations for two hundred lepers, and is already crowded, many applicants being refused admission. The enormous increase, especially during the last two decades, is believed to be largely due to preventable cause, and one for which the medical department of the English, Indian, and colonial governments cannot be freed from responsibility.' In Ceylon, as I learned from personal inquiries made in the island, leprosy is extending among the native population.

"It should be observed that Jenner received thirty thousand pounds ($150,000) for his alleged discovery before vaccination had been submitted to the test of a small-pox epidemic. The first violent small-pox outbreak showed the utter inutility of vaccination and I produced evidence before the Royal Commission in 1889 of more than five hundred cases of small-pox certified by medical authorities, after vaccination, and numerous cases of injury and death due to vaccination, within six years after Jenner's pretended discovery. In 1853, a law was passed in England making vaccination compulsory, and in 1867 the law was made more rigorous by a further act of Parliament, from which time there has been a continued and greatly increased opposition to both vaccination and compulsion up to the time when the Royal Vaccination Commission was appointed in 1889."

In *The Recrudescence of Leprosy*, Mr. Tebb gave to the world the results of his investigations mainly into one of the mischief-producing consequences of vaccination. He has published numerous other writings upon the subject, and has been one of the most active agents in spreading a knowledge of sanitation, that is, personal and municipal cleanliness, as the true prophylactic against zymotic disease, and especially small-pox. Without any acknowledgment to Mr. Tebb, boards of health have availed themselves of his labors in this respect, all the while denouncing their author, whose facts and arguments they were unable to dispute, while they relentlessly forced vaccine poison into the blood of the people.

In 1870, Mr. Tebb established in London *The Vaccination Inquirer and Health Review*, a monthly publication which became the organ of the London Society for the Abolition of Compulsory Vaccination upon its establishment shortly after. He appointed Mr. Wm. White its editor, than whom no more fortunate selection could have been made.

Mr. White wrote a masterly treatise entitled *The Story of a Great Delusion*, in which he dragged into light the entire history of this revolting superstition.

In the same year Mr. Tebb organized and contributed largely to the success of the Frst International Anti-Vaccination Congress held in Paris. A deputation waited upon the French Minister of the Interior (M. Constans), the late lamented Dr. Hubert Boëns, of Brussels, and Prof. Vogt, of Berne, and Mr. Tebb proved to the minister the many lamentable results of vaccination; and as a consequence of the facts submitted to him the bill for universal compulsory vaccination which M. Liouville had presented to the French Assembly was withdrawn. A similar deputation had audience with M. Tirard, afterwards Prime Minister. Unfortunately for France, however, medical ignorance had for a long time succeeded in thrusting the practice of vaccination upon the army and navy, and, as the entire adult male population of France (with certain unimportant exceptions) pass through either the army or navy, the result is that nearly every adult male in France is vaccinated. Vaccination is also obligatory in the public schools.

